# Exercise alters mouse sperm small noncoding RNAs and induces a transgenerational modification of male offspring conditioned fear and anxiety

**DOI:** 10.1038/tp.2017.82

**Published:** 2017-05-02

**Authors:** A K Short, S Yeshurun, R Powell, V M Perreau, A Fox, J H Kim, T Y Pang, A J Hannan

**Affiliations:** 1 Florey Institute of Neuroscience and Mental Health, Melbourne Brain Centre, University of Melbourne, Parkville, VIC, Australia; 2 Department of Pediatrics and Anatomy/Neurobiology, University of California-Irvine, Irvine, CA, USA; 3 Department of Anatomy and Neuroscience, University of Melbourne, Parkville, VIC, Australia

## Abstract

There is growing evidence that the preconceptual lifestyle and other environmental exposures of a father can significantly alter the physiological and behavioral phenotypes of their children. We and others have shown that paternal preconception stress, regardless of whether the stress was experienced during early-life or adulthood, results in offspring with altered anxiety and depression-related behaviors, attributed to hypothalamic–pituitary–adrenal axis dysregulation. The transgenerational response to paternal preconceptual stress is believed to be mediated by sperm-borne small noncoding RNAs, specifically microRNAs. As physical activity confers physical and mental health benefits for the individual, we used a model of voluntary wheel-running and investigated the transgenerational response to paternal exercise. We found that male offspring of runners had suppressed reinstatement of juvenile fear memory, and reduced anxiety in the light–dark apparatus during adulthood. No changes in these affective behaviors were observed in female offspring. We were surprised to find that running had a limited impact on sperm-borne microRNAs. The levels of three unique microRNAs (miR-19b, miR-455 and miR-133a) were found to be altered in the sperm of runners. In addition, we discovered that the levels of two species of tRNA-derived RNAs (tDRs)—tRNA-Gly and tRNA-Pro—were also altered by running. Taken together, we believe this is the first evidence that paternal exercise is associated with an anxiolytic behavioral phenotype of male offspring and altered levels of small noncoding RNAs in sperm. These small noncoding RNAs are known to have an impact on post-transcriptional gene regulation and can thus change the developmental trajectory of offspring brains and associated affective behaviors.

## Introduction

Current evidence strongly supports a role for physical exercise in the maintenance of our physical health and well being, as well as sustained mental health and cognitive capacity. There have been numerous clinical studies consistently demonstrating the broad benefits of exercise on mental health.^1, 2, 3, 4, 5^ One population-based study found that individuals who regularly exercise more than 60 min a week were less anxious or depressed than those who did not regularly exercise within their age group.^3^ A meta-analysis of exercise and anxiety studies reported that 10 weeks of aerobic exercise totaling at least 21 min per week significantly reduces anxiety traits.^4^ A recent Cochrane review is also supportive of an antidepressive effect of exercise.^6^

An individual’s engagement in physical exercise also has an impact on their offspring. Maternal physical activity of different intensities during the antenatal period modulates a child’s birthweight, neonatal adiposity and bone mineral content.^7^ Preclinical studies examining the effect of maternal exercise during pregnancy indicate that beneficial effects can be passed on to offspring.^8^ For example, pregnant rats that engaged in voluntary wheel-running and treadmill running throughout pregnancy have offspring with improved spatial learning, decreased anxiety-like behaviors and increased hippocampal brain-derived neurotrophic factor (BDNF) expression.^9, 10^ However, it remains unclear whether the effects result from physiological changes to the *in utero* environment and/or epigenetic modifications to the developing embryo.

The relevance of the paternal environment to offspring phenotypes has gained prominence in recent years with the discovery of transgenerational influence of paternal lifestyle factors on offspring phenotypes. Preclinical evidence indicates that paternal high-fat diet modeling male obesity impairs embryo development and implantation^11^ and the two subsequent generations of offspring develop increased adiposity, insulin resistance and disturbed reproductive health.^12, 13, 14, 15^ These transgenerational influences are believed to involve sperm DNA damage^16^ and altered expression levels of sperm microRNAs (miRNAs).^13^ Importantly, these can be normalized by the provision of voluntary wheel-running.^17^ In addition, exercise rescues paternal obesity-associated impairments of embryonic development and fetal growth, offspring metabolic syndrome.^17, 18^

The potential for paternal physical exercise alone to exert transgenerational effects is only beginning to be examined. It has been claimed that spatial memory of male offspring can be improved by 6 weeks of paternal forced treadmill running in C57Bl/6 mice.^19^ However, that study did not examine female offspring and is yet to be replicated. Furthermore, forced treadmill running is highly stressful; therefore, the findings may reflect paternal stress rather than paternal exercise *per se*. Finally, the offspring of control fathers exhibited below-chance performance on the Morris water maze probe trial, thus bringing those findings into question. Nevertheless, paternal exercise could influence offspring susceptibility for disease, as evidenced by the abnormal fat accumulation and weight gain observed in offspring of male mice that ran for a 12-week period prior to mating.^20^

Our laboratory has previously reported on the transgenerational influence of paternal stress on offspring anxiety, in association with altered levels of sperm small noncoding RNAs.^21^ In the present study, we hypothesized that paternal voluntary wheel-running (PR) would (1) alter anxiety- and depression-related behaviors of progeny and (2) alter the small noncoding RNA content of sperm. We found that paternal exercise was associated with the male offspring displaying more robust extinction memory as juveniles and reduced anxiety during adulthood. Small RNA sequencing identified miRNAs and transfer-derived RNAs with altered expression in the sperm of runners compared to standard-housed controls. Bioinformatics analysis identified the key biochemical signaling and cellular plasticity pathways that are targeted by miR-19b, miR-133a and miR-455. Our findings, to our knowledge, are the first lines of evidence that paternal exercise exerts a transgenerational influence on offspring emotional health in association with altered sperm small RNA content.

## Materials and methods

### Mice

All F0 C57BL/6 breeders were purchased from the Animal Resources Centre (Murdoch, WA, Australia) and habituated to the core animal facility in standard open-top laboratory mouse cages (15 × 30 × 12 cm) for 2 weeks prior to use. Standard housing comprises cages lined with sawdust and two tissues for nesting. Food and water access was *ad libitum*. Cage changes were conducted weekly. The temperature and humidity of the holding room were regulated at 22 °C and 45%, respectively. Mice were maintained on a 12 h light/dark cycle (lights on at 0700 hours). After 2 weeks of habituation, male mice were randomly allocated to 4 weeks of single housing with access to a running wheel (12 cm diameter; Pat RW group). Multiple cohorts of mice consistently ran ~50–60 km per week over the 4 weeks (data not shown). Controls were also single-housed with no opportunity to engage in running. Male F0s were then pair-mated with a naive 10-week-old C57Bl/6 female in the absence of a running wheel. Females were checked for vaginal plugs each morning. Upon observation of a vaginal plug or after 4 days, female mice were separated. F0 males resumed exercise for 1 week prior to behavioral testing. Female mice were single-housed until they littered down. F1 offspring were tested as juveniles or weaned at 4 weeks of age into open-top cages of three to four mice per cage. All experiments were approved by the Florey Institute of Neuroscience and Mental Health Animal Ethics Committee and performed in accordance with the animal research guidelines of the National Health and Medical Research Council.

### Behavioral testing

Behavioral testing of F0 males was conducted after paired matings and resumption of wheel-running for at least 1 week. Separate cohorts of postnatal day (PND) 15 and 8-week-old adult F1 offspring were tested. All behavioral testings were performed during the light phase between 0900 and 1200 hours. Mice were acclimatized to the testing room for at least 1 h before commencement of each test except for fear conditioning. Behavioral tests were conducted on separate days.

*Maternal behavior* toward the pups was observed daily from PND 0–7 and scored as described previously.^21^

*Ultrasonic vocalizations* (USVs) were recorded from PND3 pups as previously described.^21^ USV analysis was performed using the Avisoft Sound Analysis and Synthesis Software pro (Avisoft Bioacoustics, Glienicke, Germany) using the automatic detection settings, with each call manually confirmed in accordance with previous studies.^22^ The number of USV calls made per minute over the 5-min separation period was determined. Where a call could not be differentiated from background noise, the animal was excluded from analysis.

*Elevated-plus maze* testing was conducted in a room with dimmed lighting (175 lux). Movement of mice within a light-colored Perspex maze with two open arms (5 × 30 cm) and two enclosed arms (5 × 30 × 14 cm) was tracked using TopScan tracking software (CleverSys, Reston, VA, USA) over a 5-min period. Total time spent in the open arms of the maze was expressed as a percentage of the test duration. Mice were excluded from analysis if they jumped off the maze.

*Light–dark test* was conducted using locomotor cells (ENV-510, Med Associates, Fairfax, VT, USA) fitted with a dark box insert (ENV-511, Med Associates). The light half had a luminance of 700 lux. Mice started each 10-min test session in the dark half. Time spent in each half was automatically tabulated using the activity monitor software (Med Associates). Total time spent in the light half of the apparatus was expressed as a percentage of the test duration.

*Forced-swim test* was used as a measure of depression-related behavior as previously described.^23^ Briefly, 2.5 l Perspex beakers were filled with water (23–25 °C) and mice were placed inside for 5 min. Each test session was video-recorded. Total immobility time over the last 240 s was manually scored by an experienced experimenter (AS), and independently verified by a second experimenter (TP). The Depression Scan software (CleverSys) was used to automatically score the immobility time. The data were verified against times manually scored by an experienced experimenter (TP) using regression analysis, and where scores lay outside 99% confidence interval that mouse was excluded.

#### Fear conditioning

Separate groups of behaviorally naive mice were subject to fear conditioning. F1 offspring at PND15 ± 1 day and 8-week-old F1 offspring were tested as previously described.^24^ Chambers were rectangular (31.8 × 25.4 × 26.7 cm; Med Associates), with stainless steel grid floors with 36 rods (3.2 × 7.9 mm), equipped with a Med Associates VideoFreeze system (Med Associates). Two different contexts were created as described previously.^24^ A constant-current shock generator was used to deliver electric shock (0.7 mA, 1 s; unconditioned stimulus, US) to the floor of the chambers as required. A programmable tone generator, speaker and sound calibration package was used to deliver auditory tone (volume: 80 dB; frequency: 5000 Hz; conditioned stimulus, CS).

During conditioning, mice were placed in an experimental chamber for 790 s in total. During the first 2-min period, baseline freezing measurement was collected. Following this, all animals received six tone-foot-shock (CS–US) pairings. Each pairing consisted of a 10 s tone that co-terminated with a 1 s shock. Intertrial intervals ranged from 85 to 135 s, with an average of 110 s. Freezing was calculated from the first 9 s of each CS presentation to avoid confounding effects of the shock presentation on movement.^24^ Two minutes following the last presentation, the mouse was removed from the experimental chamber and placed back into the home cage.

The day following conditioning, mice were tested for their CS memory by being placed in a different context to that in which they were conditioned. Mice were allowed a 2 min period, during which baseline freezing was measured. They then received 45 presentations of a 10 s tone in the absence of the shock with intertrial interval of 10 s (that is, extinction). Percentage freezing reported is based on 10 s of tone blocked into average freezing of 15 tones to represent early, middle and late extinction. Two-week-old mice received 2 days of extinction because of high levels of freezing.

The day after extinction the mice were placed into the extinction context for 3 min and received a single reminder shock (0.7 mA) without presentation of the tone. Twenty-four hours following the reminder shock, the animals were tested for reinstatement in the same context. Mice were allowed a 2 min period, during which baseline freezing was measured. They then received 45 presentations of a 10 s tone in the absence of the shock with intertrial interval of 10 s. Data presented are representative of the first 15 presentations of the tone.

### Small RNA extraction and Illumina sequencing

For small RNA sequencing, mature spermatozoa were collected using the swim-up method.^25^ For each treatment group, three pools each consisting of sperm collected from four individual mice were collected (that is, total 12 animals per treatment group). Samples were homogenized using QIAzol lysis reagent (QIAGEN, Melbourne, VIC, Australia). Quantification and quality control were performed on the total RNA using the Agilent Bioanalyzer 2100 with the Small RNA Kit (#5067-1548, Life Technologies, Sydney, NSW, Australia). Library preparation and sequencing were performed using the Illumina HiSeq2000 workflow (Australian Genome Research Facility). Total RNA (500 ng) was used for library prep with 15 cycles of polymerase chain reaction (PCR). Quality control was performed following complementary DNA conversion using Agilent 2100 Bioanalyzer (Agilent Technologies, Melbourne, VIC, Australia). All samples were run on a single flow cell lane using 50 bp single-end reads. Following removal of dimer and adapter sequences, this was adjusted to between 2.3 and 7.7 M reads. Validation of the sequencing results was performed on sperm samples collected from five control and five running mice (not pooled).

### Real-time semi-quantitative PCR

Total RNA (1000 ng) was reverse-transcribed using miScript II RT kit (QIAGEN) according to the manufacturer’s instructions. Reverse transcription PCR was performed in a thermal cycler (E-1448, TaKaRa BIO, Otsu, Japan) under the following conditions: 37 °C for 60 min, 95 °C for 5 min. Samples were diluted 10-fold in RNase-free water and then stored at −20 °C until required. Targeted miRNA expression was determined using miScript SYBR green PCR kit (Cat #218073, QIAGEN) in conjunction with miScript Primer assays. Reactions were performed in a ViiA 7 Real-time PCR system (Applied Biosystems, Foster City, CA, USA). miRNA expression was analyzed using the comparative Ct (ΔΔCt) method and normalized to the mean expression of the control group. SNORD95 was used as the endogenous control fore sperm samples as previously reported.^21^

### Sequencing analysis

Read quality was assessed using FastQC and were trimmed against known common Illumina adapter/primer sequences using trimmomatic.^26^ All reads were then aligned to mouse genome mm10 using the Subjunc aligner within the Subread package.^27^ Sequencing data were then summarized into Reads per transcript using Featurecounts^28^ and the Gencode M2 gene models for the mouse mm10 genome build (July 2013 freeze).^29^ Normalization and statistical analysis were executed using EdgeR.^30, 31, 32^

### Functional annotation and gene ontology analyses

For analysis of predicted targets only the functionally described small RNAs were used. Initially, small RNA transcripts that were significantly increased by log2 fold change of 2 or more were included. From that list the top 20 significantly changed small RNAs were inputted into the miRWalk program.^33^ To obtain a list of downstream gene targets of the miRNAs, miRWalk 2.0 Validated Target Module was used, limited to only genes returned by miRWalk, miRanda, miRDB and Targetscan. Functional annotation of the gene list was performed using DAVID v6.7 in order to map genes to annotated KEGG pathways.^34^ The list of validated miR-interacting genes was further subject to gene ontology analysis using PANTHER Overrepresentation test version 11.0 (Released 2016-07-15)^35^ to determine classifications based on protein–protein interactions (pathways) or biological processes at the level of the cell or organism (for example, synaptic transmission). To map, name and quantify tRNA-derived RNAs (tDRs), we analyzed the trimmed reads by using tDRmapper.^36^ To predict tDR-targeted genes, we used TargetScan v5.0, which allowed us to enter custom small RNA sequences two to eight nucleotides in length (http://www.targetscan.org/vert_50/seedmatch.html). We further used *Enrichr* (http://amp.pharm.mssm.edu/Enrichr/)^37, 38^ in order to determine significantly enriched ontologies of *MGI Mammalian Phenotype Level 4* (http://www.informatics.jax.org/)^39^ of both tDRs and miRNA-targeted genes.

### Statistical analysis

Student’s *t*-tests (unpaired) were used to determine differences between two means where appropriate. Analyses of variance were used to examine main effects (for example, paternal running treatment) and/or interactions. Repeated-measures analyses of variance (ANOVAs) were used to analyze data collected from multiple time points (for example, USVs, maternal observations). Bonferroni *post hoc* tests were used to determine specific differences in the event of significant interactions. Significance was set at *P*<0.05. Sequencing data were corrected for false discovery rate (*P*<0.1). Benjamini–Hochberg false discovery rate (*q*<0.1) was applied to tDR data by converting *P*-values to *q*-values. Statistical analyses were performed using SPSS statistics version 22.0 (IBM, Armonk, NY, USA) and GraphPad prism 6.0 (GraphPad software, LA Jolla, CA, USA). All graphs presented as mean±s.e.m.

## Results

### Running did not alter male behavior, mating parameters or maternal care

No significant effect of running was detected by the elevated-plus maze for time in the open arms (*t*_(18)_=0.343, *P*=0.736) and total distance traveled (*t*_(__18)_=1.14, *P*=0.270). Similarly, there was no difference between the groups in the light–dark box for total time in the light half (*t*_(18)_=0.443, *P*=0.663) or exploratory distance (*t*_(18)_=1.54, *P*=0.141). There was no effect of exercise on the total immobility time (*t*_(18)_=1.54*, P*=0.140) nor the latency to the first bout of immobility (*t*_(18)_=0.6912, *P*=0.498) in the forced-swim test. There was no effect of running on the success rate of paired matings or on litter sizes (data not shown). Postpartum maternal behavior (nurturing, neglect, self-maintenance) was scored twice daily for the first 7 days with no differences observed (data not shown).

### Male offspring conceived following paternal exercise do not reinstate conditioned fear memory as juveniles and have reduced anxiety as adults

**Figure 1:**
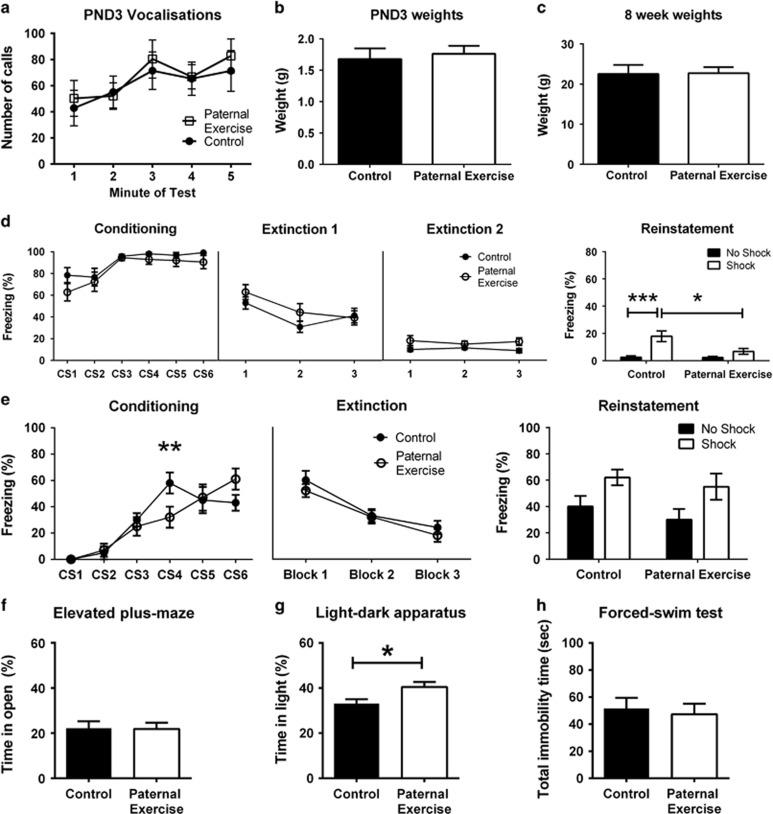
Transgenerational response of male offspring to paternal exercise. Ultrasonic vocalizations recorded over a 5 min separation period at postnatal day 3 (**a**; *n*=10 per group). Offspring body weights at postnatal day 3 (**b**) and 8 weeks of age (**c**). Freezing response of 2-week-old offspring during fear conditioning, extinction and reinstatement (**d**; *n*=18 per group for conditioning and extinction, *n*=8–10 per group for reinstatement). A separate cohort of 8-week-old offspring was used for fear memory testing (**e**; *n*=15–22 per group for conditioning and extinction, *n*=7–11 per group for reinstatement. Anxiety assessed on the elevated-plus maze (**f**; *n*=25–27 per group) and light–dark apparatus (**g**; *n*=25–27 per group). Depression-related behavior assessed with the forced-swim test (**h**; *n*=25–27 per group). *P<0.05, **P<0.01, ***P<0.001. Data presented as group mean±s.e.m.

No significant differences were detected between paternal exercise conditions for the overall number of vocalizations made by male pups (F_(1,16)_=0.150, *P*=0.706; Figure 1a). There was an overall effect of time (F_(4,64)_=3.83, *P*=0.0075) but no paternal treatment × time interaction (F_(4,64)_=0.180, *P*=0.946). The body weights of PND3 pups did not significantly differ between groups (*P*=0.323; Figure 1b). Male offspring also did not differ in body weights at 8 weeks of age (*P*=0.804; Figure 1c).

At 2 weeks of age, there was no effect of paternal exercise during fear conditioning in male offspring (F_(1,34)_=2.68, *P*=0.111), with all animals learning to associate the tone with the foot shock over time (F_(5,170)_=10.2, *P*<0.001; Figure 1b), but no treatment × time interaction (F_(5,170)_=0.480, *P*=0.793). Freezing significantly reduced across extinction trials Day 1 (F_(2,68)_=10.1, *P*<0.001) but there was no overall effect of paternal treatment (F_(1,34)_=0.950, *P*=0.337), and no significant trial × paternal treatment interaction (F_(2,68)_=1.38, *P*=0.259). Freezing levels did not change during Day 2 (F_(2,68)_=0.11, *P*=0.892); however, there was a trend toward an overall effect of paternal treatment (F_(1,34)_=4.06, *P*=0.0518). There was no significant trial × paternal treatment interaction for Day 2 data (F_(2,68)_=0.870, *P*=0.425). The day after extinction, in order to assess reinstatement, half the mice then received a single reminder shock in the extinction context, and freezing was recorded the next day. There was a significant effect of reminder shock (F_(1,32)_=17.48, *P*=0.0002), a significant effect of paternal treatment (F_(1,32)_=5.67, *P*=0.0234) and a shock × paternal treatment interaction (F_(1,32)_=5.3, *P*=0.0279). Owing to the significant interaction, *post hoc* tests were carried out per factor, which revealed a significant increase in freezing following the reminder shock in control animals (*P*<0.001), but not in PR offspring (*P*>0.05).

At 8 weeks of age, fear conditioning of male offspring was not affected by paternal treatment (F_(5,175)_=0.360, *P*=0.552; Figure 1c). As expected, overall freezing behavior increased with repeated CS trials (F_(5,175)_=53.5, *P*<0.001) and there was a significant paternal exercise × CS interaction (F_(5,175)_=4.07, *P*=0.00160). *Post hoc* analysis of this interaction revealed a significant difference between the groups only in the fourth CS–US trial (*t*_(210)_=3.52, *P*=0.00320), with both groups showing comparable freezing by the sixth presentation of the CS (*t*_(210)_=2.08, *P*=0.230). During extinction, there was an overall effect of trial (F_(2,70)_=60.8, *P*<0.001), but no effect of paternal exercise (F_(1,35)_=0.640, *P*=0.429), nor a paternal exercise × trial interaction (F_(2,70)_=0.450, *P*=0.641). Reinstatement was also assessed in 8-week-old offspring. There was an overall effect of shock (F_(1,31)_=7.54), *P*=0.0100), but no effect of paternal exercise (F_(1,31)_=2.08, *P*=0.160), nor a paternal exercise × shock interaction (F_(1,31)_<0.01, *P*=0.995).

No differences were observed in the time spent exploring the open arms of the elevated-plus maze (*t*_(50)_=0.0856, *P*=0.932; Figure 1d) and distance traveled (*t*_(50)_=0.596, *P*=0.554; data not shown). In the light–dark box, male PR offspring spent significantly more time in the light zone (*t*_(50)_=2.49, *P*=0.0161; Figure 1e) and recorded greater distance moved (*t*_(50)_=2.21, *P*=0.0319; data not shown). In the forced-swim test, there was no effect of paternal treatment on immobility time (*t*_(50)_=0.394, *P*=0.696; Figure 1f) and latency to the first bout of immobility (*t*_(50)_=0.457, *P*=0.650; data not shown).

### No transgenerational effects of paternal running on female offspring conditioned fear and anxiety

**Figure 2:**
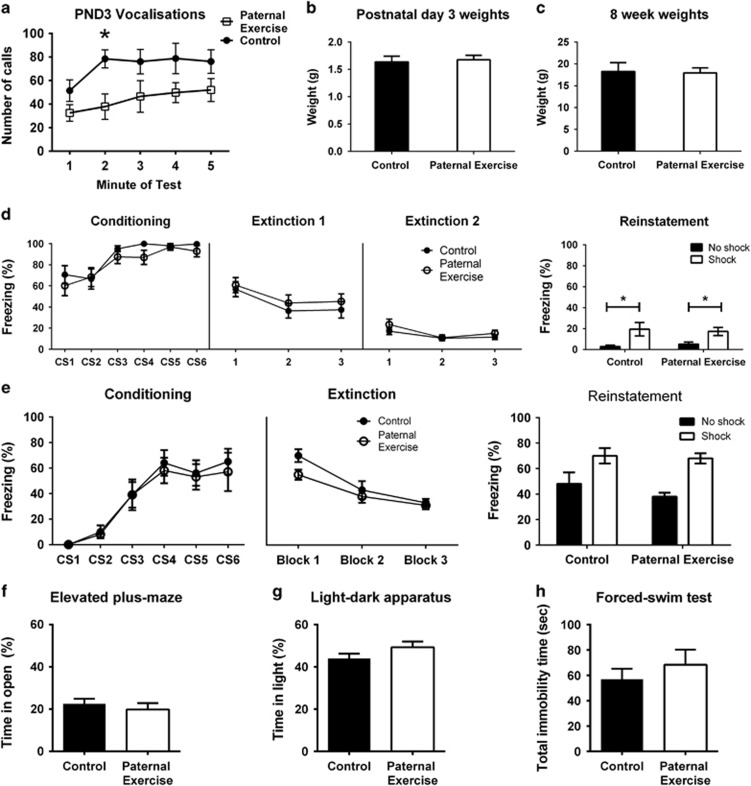
Transgenerational response of female offspring to paternal exercise. Ultrasonic vocalizations recorded over a 5 min separation period at postnatal day 3 (**a**; *n*=8). Offspring body weights at postnatal day 3 (**b**) and 8 weeks of age (**c**). Freezing response of 2-week-old offspring during fear conditioning, extinction and reinstatement (**d**; *n*=17–19 per group for conditioning and extinction, *n*=8–10 per group for reinstatement). A separate cohort of 8-week-old offspring was used for fear memory testing (**e**; *n*=13 per group for conditioning and extinction, *n*=6–7 per group for reinstatement). Anxiety assessed on the elevated-plus maze (**f**; *n*=19–35 per group) and light–dark apparatus (**g**; *n*=19–35 per group). Depression-related behavior assessed with the forced-swim test (**h**; *n*=19–35 per group). *Post hoc t*-test: *P<0.05. Data presented as group mean±s.e.m.

In contrast to male pups, there was a significant difference in the overall number of USVs made by female PR offspring (F_(1,17)_=9.62, *P*=0.0065; Figure 2a), indicating that paternal exercise decreases USVs in female pups. No overall effect of time on the number of calls made (F_(4,68)_=2.07, *P*=0.0944) and no paternal treatment × time interaction (F_(4,68)_=0.390, *P*=0.818). There was no difference in pup body weight at PND3 (*P*=0.475; Figure 2b). There was also no difference in body weights at 8 weeks of age (*P*=0.570; Figure 2c).

Juvenile female offspring showed increased freezing across conditioning (F_(5,170)_=13.38, *P*<0.001; Figure 2d), with no effect of paternal treatment (F_(1,34)_=1.80, *P*=0.189) nor a trial × paternal treatment interaction (F_(5,170)_=0.480, *P*=0.794). For extinction, CS-elicited freezing decreased across trials on day 1 (F_(2,68)_=8.37, *P*=0.0006) and day 2 (F_(2,68)_=6.07, *P*=0.0037), but no other effects of interactions on those days were observed. Following reinstatement, there was a significant effect of reminder shock (F_(1,32)_=13.3, *P*=0.0009), but no effect of paternal treatment (F_(1,32)_=0.0004, *P*=0.984) nor a shock × paternal treatment interaction (F_(1,32)_=0.330, *P*=0.570).

At 8 weeks of age, female offspring showed increased freezing across conditioning trials (F_(5,120)_=36.3, *P*<0.0001; Figure 2e), but no effect of paternal treatment (F_(1,24)_=0.350, *P*=0.559), nor a time × paternal treatment interaction (F_(5,120)_=0.120, *P*=0.987). Freezing also significantly decreased over extinction trials (F_(2,48)_=45.4, *P*<0.0001), without any effects of paternal treatment (F_(1,22)_=1.64, *P*=0.180), or trial × paternal treatment interaction (F_(2,48)_=1.71, *P*<0.193). Following reinstatement, there was a significant effect of shock presentation (F_(1,22)_=19.0, *P*=0.0002) but no effect of paternal treatment (F_(1,22)_=1.64, *P*=0.213) nor a shock × paternal treatment interaction (F_(1,22)_=0.600 m, *P*=0.445).

No differences were detected in the elevated-plus maze based on time spent in the open arms (*t*_(52)_=0.695, *P*=0.490; Figure 2f) and total distance traveled (*t*_(52)_=0.745, *P*=0.456; data not shown). In the light–dark box, no differences were observed in the time spent in the light zone (*t*_(51)_=1.45, *P*=0.154; Figure 2g) and total distance traveled (*t*_(51)_=0.0084, *P*=0.993; data not shown). In the forced-swim test, no differences were found for total immobility time (*t*_(52)_=0.804, *P*=0.425; Figure 2h) and latency to first bout of immobility (*t*_(52)_=0.0136, *P*=0.989; data not shown).

### Running alters miRNA and tRNA content of sperm

**Figure 3:**
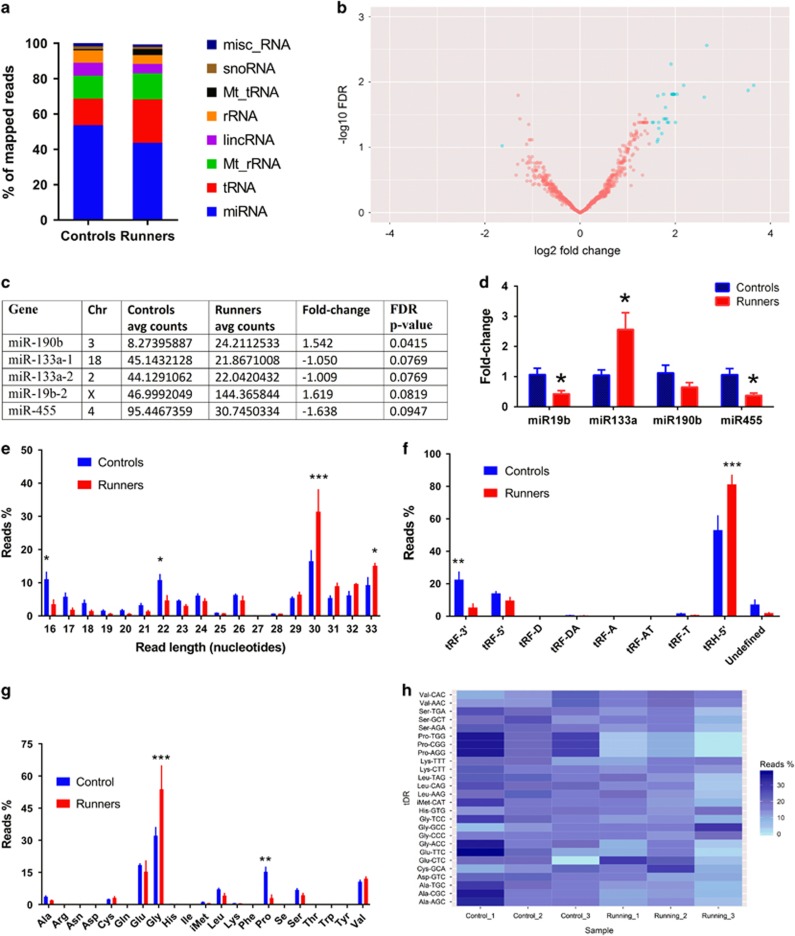
Running-induced changes to microRNAs (miRNAs) and tRNA-derived fragments revealed by small RNA sequencing of sperm. Small RNA biotype from genomic annotation presented as percentage of the total mapped reads (**a**). Volcano plot depicting the fold changes in small RNAs as being differentially expressed in the sperm of runners compared with controls (**b**). Blue dots represent small RNAs that are significantly altered (false discovery rate, FDR<0.1) with log fold change of more than 1.5. List of the significantly altered miRNAs (**c**); note that 133a-1 and 133a-2 map to different locations in the genome. An independent group of animals was used to conduct quantitative PCR (qPCR) validation of the differentially expressed miRNAs (**d**). Size distribution of tRNA-derived RNA fragments (tDRs) in the sperm (**e**). Classification of tDRs by type (**f**). Amino acid carry distribution of tDRs (**g**). Heatmap of tDRs in each sample by their anticodon profile (**h**). FDR: *q <0.1, **q<0.01, ***q<0.001.

Small RNA sequencing of sperm collected from wheel-running and control mice yielded a total of 849 mapped genes (Figure 3a). After correction for false discovery rate, 84 genes (9.9%) were differentially expressed between runners and controls (Figure 3b). The expression of 76 genes (9.0%) was increased in the sperm of runners, while 8 genes (0.9%) had decreased expression. Only four miRNAs were differentially expressed in runners (Figure 3c). miR-190b and miR-19b-2 were increased and miR-133a-1, miR-133a-2 (same gene sequences mapped to different chromosomes) and miR-455 were decreased. We confirmed that running resulted in differential expression of three of the four miRNAs by validating these candidate genes using RNA isolated from a separate group of running and control mice (Figure 3d). We confirmed that miR-455 was significantly decreased (*t*_(8)_=3.16, *P*=0.0134) in sperm of runners. Surprisingly, miR-133a was upregulated (*t*_(8)_=2.59, *P*=0.0320) and miR-19b was downregulated (*t*_(8)_=2.75, *P*=0.0250) in the validation study. There was a 40% decrease in miR-190b, but this was not significant (*t*_(8)_=1.59, *P*=0.151).

In addition to differentially expressed miRNAs, we discovered that of 299 genes that mapped for tRNAs, 51 (17%) were significantly altered—all increased. Small RNAs of 29–33 nt are 5′ tRNA halves (5′-tRH) and those of 16–22 nt are tRNA fragments (tRF). Thus, we probed the data to determine the contribution of tRFs with different nucleotide lengths. We found that 16 nt (*q*=0.012) and 22 nt (*q*=0.0498) fragments were significantly reduced, while 30 nt (*q*<0.001) and 33 nt (*q*=0.052) fragments were significantly increased (Figure 3e). This corresponded to significantly increased levels of 5′-tRHs (*q*<0.001) and significantly decreased levels of 3′-tRFs (*q*=0.001) in the sperm of runners compared to controls (Figure 3f). We further looked into the species of tDRs by separating them into the amino acid they carry and their anticodon sequences. This analysis showed significantly elevated levels of tDRs that carry Gly (5′-tRH; *q*<0.001) and significantly reduced levels of tDRs that carry Pro (3′-tRF; *q*=0.001; Figure 3g). Furthermore, among the altered tRNA-derived fragments, only tRNA-Gly-GCC was significantly altered from multiple comparisons of 45 different anticodons (*q*<0.001; Figure 3h).

### Functional annotation and gene ontology analysis

**Figure 4:**
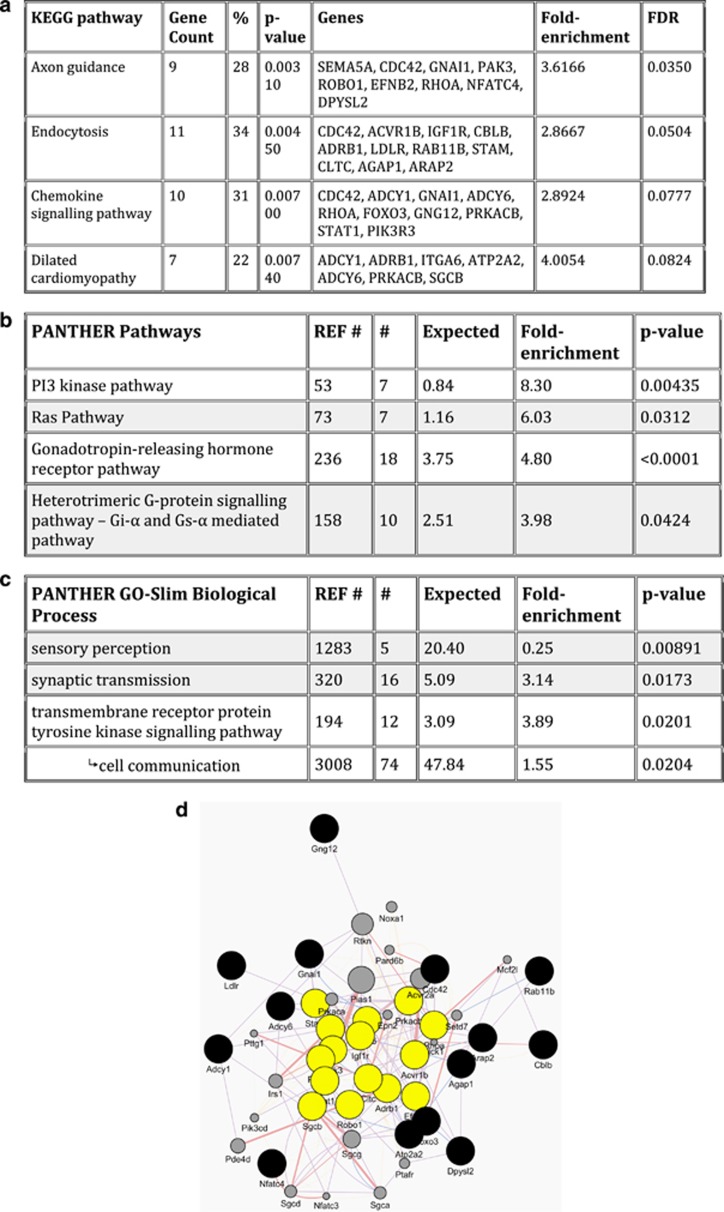
Functional annotation and gene ontology based on differentially expressed miRNAs in sperm. Functional annotation of the downstream target genes of the differentially expressed miRNAs that were validated revealed over-representation of four pathways (**a**). Gene ontology analysis revealed four significantly represented molecular signaling pathways (**b**), related to three biological classifications (**c**). A diagrammatic representation of the molecular network connectivity based on the four over-represented KEGG pathways (**d**).

On the basis of the three miRNAs that were validated to be differentially regulated by running, and as most miRNAs have pleiotropic functions, we used the miRWalk 2.0 Validated Target Module to obtain a list of 355 unique and validated downstream gene targets. Only one gene—trinucleotide repeat-containing gene 6B, *Tnrc6b*—was the common target of two or more miRNAs (miR-19b and miR-133a). DAVID analysis identified pathways regulated by the gene targets, revealing 24 annotated KEGG pathways (Supplementary Table 1), four of which passed corrections for false discovery rate (Figure 4a). These were axon guidance (fold-enrichment 3.6, *P*=0.0350), endocytosis (fold-enrichment 2.9, *P*=0.050), chemokine signaling pathway (fold-enrichment 2.9, *P*=0.0777) and dilated cardiomyopathy (fold-enrichment 4.0, *P*=0.0824).

We used gene ontology PANTHER analysis to identify relationships between all the predicted target genes of the three miRNAs. Enriched pathways predicted to be regulated were the GnRH receptor pathway (fold-enrichment 4.8, *P*<0.001), phosphatidylinositol 3 kinase (fold-enrichment 8.3, *P*=0.00435) and Ras-signaling pathways (fold-enrichment 6.0, *P*=0.0312), as well as the heterotrimeric G-protein signaling pathway (fold-enrichment 3.98, *P*=0.0424; Figure 4b). When the gene list was sorted by biological processes, there was over-representation of biochemical pathways related to sensory perception (fold-enrichment 0.25, *P*=0.00891), synaptic transmission (fold-enrichment 3.14, *P*=0.0173) and Trk-signaling pathway (fold-enrichment 3.89, *P*=0.0201; Figure 4c). Further categorization by molecular function revealed significant over-representation of G-protein modulators (20/446; *P*=0.00849), with the majority being guanyl-nucleotide exchange factors (including members of the Ras super family GTPases).

We conducted a GeneMANIA analysis using a limited list of the 29 unique genes implicated in the four significantly represented KEGG pathways based on co-expression, physical interactions and co-localization (Figure 4d). Interestingly, we noted similarities with the gene ontology analysis with the central cluster including elements of phosphatidylinositol 3 kinase-signaling (*Pik3r3, Pik3ca* and *Pik3cd*), Ras superfamily signaling (*Rhoa, Cdc42, Rab11b, Pak3* and *Rtkn*), G-protein-coupled receptors (β1-adrenergic receptor, *Adrb1* and insulin-like growth factor receptor 1, *Igf1r*) and regulators of cytoskeletal dynamics (*Cltn, Stat1, Pias, Sgcb, Efnb2* and *Robo1*).

Comparison of the predicted gene targets of the top-ranked altered tDRs Gly(C/G)CC (only 5′-tRHs have a similar role and function as miRNAs) was generated by custom TargetScan. The predicted gene targets of the miRNAs and tDRs targets revealed no commonalities, suggesting that the miRNA and tRNAs have distinct downstream gene targets. Comparison of both gene lists against the Mouse Genome Informatics database by Enrichr generated an expanded scope of regulated pathways, revealing 118 potential significant ontologies for the miRNA target list and 41 significant ontologies for the Gly(C/G)CC target list. Remarkably, both lists demonstrated strikingly similar ontologies related to brain function and behavior, as well as motor capabilities. There were 23 significant ontologies common to both miRNA and Gly(C/G)CC lists. Interestingly, common significant ontologies included abnormal affective behavior, learning and memory, and neuron morphology.

## Discussion

The findings of this study reveal novel transgenerational responses to voluntary wheel-running, which manifest in male offspring, and are likely to involve a running-induced alteration of small noncoding RNAs contained in sperm. We show that male offspring of runners display lower anxiety levels and more robust fear extinction memory. Running changed the miRNA and tRNA content of sperm, and we speculate that these epigenetic alterations elicit the transgenerational response observed in the male offspring. Recent work has drawn much attention to how paternal stress^21, 25, 40, 41^ and diet^13, 42, 43^ separately alter offspring phenotypes; however, the transgenerational effects of paternal physical activity have not been adequately addressed. We have provided, to our knowledge, the first preclinical evidence that paternal voluntary exercise indirectly influences offspring mental health outcomes, adding to its previously described impact on offspring physical health and disease susceptibility.^20, 44^

Epidemiological evidence indicates that regular physical activity reduces the risk for a range of diseases^45, 46, 47^ and is also important for good mental health.^48^ While the benefits for oneself are long established, the transgenerational influence of exercise on offspring behavior and mental health remains unknown. Addressing this issue in the human population would be a challenging endeavor, as such a study would have to be stringently designed in order to take into account the numerous genetic variables and non-genetic factors across both generations. Even parental attitudes toward exercise could be a significant factor, as one study of preschool-aged children has reported that paternal support for physical activity is positively correlated to their offspring’s own engagement in physical activity,^49^ which in turn influences their mental and physical health. Nonetheless, it would be interesting to attempt a prospective study of preconceptual paternal exercise and its relationship with the mental health outcomes of their children.

The collective evidence based on the few preclinical studies attempted to-date, including the present findings, indicates that parental (preconception) physical exercise alters the physical and behavioral phenotypes of offspring. However, there is some controversy pertaining to the directionality of the paternally transmitted effects, with unexpectedly detrimental effects on offspring health. Work by Murashov *et al.*^20^ found that the offspring of wheel-running mice had impaired glucose tolerance and responded to a high-fat diet with greater weight gain and fat accumulation. We did not impose any metabolic challenges on the mice in this study, as it could compromise the interpretation of their behavioral responses in the tests we conducted.^50, 51^ However, we detected no differences in offspring body weight at birth, weaning (4-week-old) and during adulthood, replicating the findings of Murashov *et al.*^20^ of their control diet-fed offspring mice. In contrast, exercise during pregnancy leads to offspring with greater birth weights (which normalize by adulthood), greater propensity for running and increased running-induced fat loss.^52, 53, 54^ The stark differences between maternal and paternal-driven metabolic outcomes require further investigation, and could be attributed to sex-linked metabolism genes. It would also be interesting for future studies to investigate whether paternal running also modulates running behavior of the F1 offspring, or if maternal running dictates the physiological response of F1 offspring to a high-fat diet. Further work will be also be required to determine whether there is any impact manifested by future generations (grand-child/F2 generation and beyond). Our results strengthen the current evidence that body weight is largely influenced by maternal factors.^55, 56, 57^

The behavioral shift observed in only the male F1 offspring could imply a protective effect against stress and its related behaviors of paternal exercise on offspring, as lower levels of anxiety confer resilience to social stressors.^58^ Sexual dimorphism is a common observation in studies of rodent behavior, particularly for affective behaviors relevant to psychiatric conditions. It would be interesting to investigate whether the F1 offspring manifest additional susceptibility for psychopathology. In searching the literature for rodent models wherein only males display altered anxiety behavior, we identified potential molecular modifiers of behavior. For example, one recent paper using optogenetic approaches to modulate the cellular activity of oxytocin-expressing interneurons in the medial prefrontal cortex discovered a strong anxiolytic effect in male mice, in contrast to female mice that displayed no anxiety-related behavioral changes.^59^

While the anxiolytic properties of running are well established by rodent studies,^60, 61, 62, 63^ the present study, to our knowledge, is the first to provide evidence of an effect on anxiety behavior transmitted via the male germ line. The transgenerational influence of paternal exercise was limited to anxiety and does not extend to behavioral responses of the depression spectrum, as we detected no difference in the forced-swim test. In addition, it was specific to the light–dark box, as no behavioral difference was detected in the elevated-plus maze. Inconsistencies between these two long established and well-validated tests of anxiety behavior are not uncommon in the literature. In fact, the discordance in behavioral responses could reflect molecular changes occurring in specific brain regions. For example, transgenic mice with a forebrain-specific disruption of calcineurin signaling spend a decreased amount of time in the light half of the light–dark box but an increased amount of time in the open arms of the elevated-plus maze.^64^ In a similar vein, forebrain-specific knockouts of the glucocorticoid receptor spend increased time in the open arms of the elevated-plus maze but do not differ in the light–dark box.^65^ The forebrain (the medial prefrontal cortex in particular) regulates innate anxiety behavior and is heavily implicated in the pathophysiology of anxiety disorders.^66, 67^ Even though our observed behavioral phenotype is not identical to other rodent models of sexually dimorphic anxiety, there is sufficient similarity to lead us to hypothesize an involvement of differential glucocorticoid receptor and/or calcineurin-dependent signaling activity in the forebrain in the male phenotype. The involvement of calcineurin potentially extends beyond anxiety behavior, as a separate study has reported that the manipulation of forebrain calcineurin activity also influences the extinction of contextual fear memory.^68^ The molecular regulation of forebrain activity should therefore be subject to further study in order to determine whether these brain regions are involved in the manifestation of the anxiolytic phenotype.^69^

Wheel-running is closely associated with increased hippocampal neurogenesis and improved cognitive performance,^70, 71, 72^ both of which are negatively influenced by social isolation stress^73, 74^ and corticosterone treatment.^75^ As running is anxiolytic for the fathers and the offspring, it would be of interest to investigate whether paternal voluntary exercise also exerts a transgenerational influence over offspring cognition and hippocampal neurogenesis.

There is still much to be discovered and understood about the underlying biological mechanisms regulating transgenerational responses. Studies of mouse models of paternal stress have revealed a role of miRNAs.^21, 25, 40^ The epigenomic profile of sperm is also subject to modification by exercise. In humans, 3 months of exercise alter DNA methylation levels in sperm;^76^ however, changes to the small noncoding RNAs have not been investigated. A recent study that provided male mice with 12 weeks of voluntary wheel-running reported changes in the expression levels of miR-483, miR-431, miR-21 and miR-221 in sperm.^20^ These miRNAs regulate developmental and metabolic gene signaling pathways, which the authors link to the resulting metabolic phenotype of offspring. We have taken an unbiased broader approach to identify the effects of exercise on the small noncoding RNA population by utilizing RNA sequencing. Interestingly, RNA sequencing did not identify any of the miRNAs targeted by Murashov *et al.*^20^ Instead, we were surprised that a minimal number of miRNAs were altered, while a much larger number of tRNAs were changed. One key difference between the present study and that of Murashov *et al.* is that we provided male breeders with 4 weeks of wheel-running compared to 12 weeks. It is unclear whether an additional 8 weeks of running confers additional changes to miRNA expression in sperm. One way to determine this could be to profile the metabolic phenotype of the offspring in our study.

Associated with these positive effects of exercise, numerous cellular and molecular changes in the brain in response to running have been described in rodents.^77^ At a molecular level, changes in gene expression have been described, including upregulation of neurotrophic factors such as vascular endothelial growth factor^78^ and BDNF.^79, 80, 81, 82^ In addition to a role in learning and memory after exercise,^83^ levels of BDNF protein have been associated with an alteration of anxiety levels following exercise.^79, 80^

Besides miRNAs, the probable regulatory role of other species of small noncoding RNAs cannot be overlooked. We found that the abundance of two subsets of tRNA-derived fragments was changed in the sperm of runners, namely a downregulation of tRF-3′ (22 nt) and an upregulation of tRH-5′ (tRNA halves, 30 and 33 nt). tRNAs are the most abundant of all small noncoding RNA molecules and are fundamental to mRNA-to-protein translation. However, tRNA-derived fragments and halves are biologically distinct (see reviews by Kirchner and Ignatova,^84^ and Keam and Hutvagner^85^) and are involved in highly conserved molecular responses to heat stress, oxidative stress and aging.^86, 87^ tRNA-derived fragments are enriched in mature mouse sperm, in particular those that are derived from tRNA-Glu and tRNA-Gly.^88^ Our results are consistent with the current literature, as tDRs from Glu and Gly tRNAs were mapped for almost 50% of the reads in the sperm of control sedentary mice. However, while the percentage of reads is almost identical for tRFs (both 3′ and 5′) and tRNA halves (5′), running resulted in an enrichment of tRNA halves, which accounted for 80% of the tRNA reads. The vast majority of these reads are tRH-5′-Gly-GCC, which were significantly elevated in the exercise group compared to controls.

We now know that tRFs also have a role in the transgenerational modification of offspring phenotypes via the male germ line. Maturing sperm in the epididymis acquire their tRNA content from exosomes secreted by epithelial cells.^89^ Chen *et al.*^90^ discovered that a high-fat diet altered the abundance of tRNA-derived fragments in mature mouse sperm, and when the subpopulation of RNAs ranging from 30 to 40 nt was injected into zygotes (at a concentration equivalent to 10 sperm), it yielded offspring with the expected metabolic pathophysiologies. Thus, we speculate that our observation of running-induced changes in the male offspring behavioral phenotype is likely to involve running-induced alterations of the abundance of tRNA-derived fragments in sperm. Chen *et al.*^90^ also discovered that RNA modifications were vital for tRH-mediated transgenerational modification of the offspring phenotype. Synthetic tRHs were less stable and degraded more quickly, and did not reproduce the same transgenerational effect. As we have found that tRF-Gly and tRF-Pro are specifically altered in sperm of the runners, these states of modification could be a focus of future investigations.

Another study of paternal low-protein diet provided further evidence that 5′-tRHs are involved in the transgenerational response by offspring.^89^ In addition, it was observed that tRH-5′-Gly-GCC is transferred to the sperm by epididymosomes, where they function as gene repressors. How interorgan dietary signals ultimately converge to modify the tRNA content of epididymosomes remains unknown. We are only beginning to understand how an altered load of tRNA in a sperm could alter embryonic development leading to a particular offspring phenotype. Embryo microinjections of tRH-Gly-GCC antisense sequences result in an upregulation of many genes that regulate embryonic development, suggesting that offspring phenotypes are a direct result of altered physiological and brain development. A recent study of paternal obesity also reported that tRH-5′-Gly-GCC was a likely candidate for the transgenerational modification of offspring metabolic phenotypes.^91^ Therefore, our findings of increased levels of tRH-5′-Gly-GCC in the sperm of runners might imply transgenerational effects on the metabolic phenotype of offspring, and is the focus of on-going studies.

In summary, exercise was found to significantly alter the small noncoding RNA content of sperm, associated with an anxiolytic behavioral phenotype of male offspring. To our knowledge, this is the first direct body of evidence that paternal exercise can induce transgenerational transmission of affective behavioral traits to their offspring. These findings suggest that male lifestyle factors are significant modifiers of offspring behavioral phenotypes, in addition to the previously established metabolic and physiological effects. It is worth speculating that phenotype reprogramming serves an evolutionary purpose to optimize the survivability of offspring under the same environmental conditions as their fathers. Although it is not yet clear whether such specific effects exist or are even measurable in humans, it has been reported that paternal engagement in physical exercise is positively associated with the development of fundamental movement skills of their preschool children;^92^ future studies could extend to children’s academic performance. Together, these results suggest that paternal physical activity is a critical epigenomic modifier that should be pursued in future transgenerational studies of humans and other animals.
