# Supplementary material for: Exercise alters mouse sperm small noncoding RNAs and induces a transgenerational modification of male offspring conditioned fear and anxiety
Source: Transl Psychiatry. 2017 May 2;7(5):e1114–. doi: 10.1038/tp.2017.82 (PMC5534950; doi:10.1038/tp.2017.82)
Supplement: Supplementary Information [file tp201782x1.docx]

| **KEGG pathway** | **Gene Count** | **%** | **p-value** | **Benjamini** |
| --- | --- | --- | --- | --- |
| Axon guidance | 9 | 2.7 | 0.00310 | 0.310 |
| Endocytosis | 11 | 3.3 | 0.00450 | 0.240 |
| Chemokine signalling pathway | 10 | 3 | 0.00700 | 0.250 |
| Dilated cardiomyopathy | 7 | 2.1 | 0.00740 | 0.200 |
| Pancreatic cancer | 6 | 1.8 | 0.01100 | 0.240 |
| Focal adhesion | 10 | 3 | 0.01200 | 0.220 |
| Pathways in cancer | 13 | 3.9 | 0.01800 | 0.270 |
| Regulation of actin cytoskeleton | 10 | 3 | 0.02000 | 0.270 |
| Progesterone-mediated oocyte maturation | 6 | 1.8 | 0.02100 | 0.250 |
| Wnt signaling pathway | 8 | 2.4 | 0.02200 | 0.230 |
| Gap junction | 6 | 1.8 | 0.02200 | 0.220 |
| T cell receptor signaling pathway | 7 | 2.1 | 0.02300 | 0.210 |
| MAPK signaling pathway | 11 | 3.3 | 0.02700 | 0.220 |
| Viral myocarditis | 6 | 1.8 | 0.03200 | 0.240 |
| Melanogenesis | 6 | 1.8 | 0.04000 | 0.280 |
| p53 signaling pathway | 5 | 1.5 | 0.04000 | 0.270 |
| Tight junction | 7 | 2.1 | 0.04100 | 0.260 |
| Adherens junction | 5 | 1.5 | 0.05400 | 0.310 |
| Oocyte meiosis | 6 | 1.8 | 0.06500 | 0.350 |
| Leukocyte transendothelial migration | 6 | 1.8 | 0.07300 | 0.370 |
| Endometrial cancer | 4 | 1.2 | 0.07400 | 0.360 |
| Purine metabolism | 7 | 2.1 | 0.07500 | 0.350 |
| Colorectal cancer | 5 | 1.5 | 0.07800 | 0.350 |
| Neurotrophin signaling pathway | 6 | 1.8 | 0.09800 | 0.410 |

**Supplementary Table 1. KEGG pathways represented by genes obtained from miRWalk Validated Target Module search (ranked by p-value).**
